# Nonsurgical management of *Fusobacterium necrophorum* sternoclavicular septic arthritis: a case report

**DOI:** 10.1186/s13256-022-03316-8

**Published:** 2022-03-03

**Authors:** SangMin Kim, Ruhi Kanwar, M. Blair Marshall

**Affiliations:** 1 Harvard Medical School, Boston, MA 02115 USA; 2 Division of Thoracic Surgery, Department of Surgery, Brigham and Women’s Hospital, Harvard Medical School, 75 Francis Street, Boston, MA 02115 USA

**Keywords:** Sternoclavicular septic arthritis, *Fusobacterium necrophorum*, Conservative management, Thoracic infections

## Abstract

**Background:**

To date, the gold-standard treatment for sternoclavicular septic arthritis has been surgery due to the high failure and complication rates of medical treatment. In particular, presentation of *Fusobacterium* sternoclavicular septic arthritis has been rarely reported and very sparsely investigated, and only one other case report of septic arthritis caused by this pathogen exists in literature.

**Case presentation:**

We report a case of an otherwise healthy 38-year-old Caucasian woman who presented with sternoclavicular septic arthritis as a complication of *Fusobacterium necrophorum* mediastinitis. Our patient underwent successful management through nonstandard, conservative treatment of 7 weeks of intravenous piperacillin + tazobactam followed by 6 weeks of oral amoxicillin + clavulanic acid.

**Conclusion:**

We highlight a case of the rare presentation of *Fusobacterium necrophorum* sternoclavicular septic arthritis that did not require surgical intervention for successful management. Though infection of the sternoclavicular joint is unusual, it continues to be seen in thoracic surgery, and there are increasing numbers of antibiotic-resistant organisms. This case broadens insight into the clinical course and treatment of such conditions. The success of conservative management in this case aligns with the similar nonsurgical course of the one previous report of *Fusobacterium* sternoclavicular septic arthritis occurrence. Thus, further discussion and thought for reevaluating the current standard practice of surgery for sternoclavicular joint infection is suggested. Our case supports assessing a patient’s overall health, causative organism, and extent of infection in interventional course and taking the feasibility of conservative management into more weighted consideration.

## Background

Sternoclavicular septic arthritis (SCSA) is a rare condition most commonly seen in IV drug users and may progress to life-threatening complications [1, 2]. Its incidence remains unknown and unusual, but mentioned within thoracic surgery [2]. *Staphylococcus aureus*, *Streptococcus* (*pneumoniae*, *pyogenes*, *viridans*), *Pseudomonas*, and *E. coli* are among the most commonly identified underlying pathogens [3–5]. *Fusobacterium necrophorum* is an obligate anaerobic, nonmotile, non-spore-forming, Gram-negative rod that is part of the normal flora of the oral cavity, female genital tract, and gastrointestinal tract [6]. The microbe is commonly implicated in Lemierre’s syndrome and following the advent of antibiotics has significantly decreased in incidence, being seen as a “forgotten disease.” However, clinical reemergence of infection and syndrome presentation is now increasingly common due to resistant organisms [7]. There are case reports of *F. necrophorum* causing severe infection in otherwise healthy patients, requiring broad-spectrum antibiotics for its treatment [8]. To our knowledge, this is the second report of *Fusobacterium necrophorum* sternoclavicular septic arthritis and the first since 1993 [9]. We aim to broaden available insight into a rare but relevant clinical condition, currently lacking presentation in literature.

Due to limited evidence given its rarity and significant risk associated (i.e., osteomyelitis) with failure of conservative treatment, therapeutic strategies to treat *F. necrophorum* SCSA varies widely across providers [11, 15]. Although conservative management has been attempted, its failure rate is as high as 83%, with serious associated complications such as osteomyelitis [11]. In general, SCSA is managed aggressively at most medical centers, and surgical resection involving muscle flap closure is considered its gold-standard treatment.

We present the case of a healthy 38-year-old women presenting with *Fusobacterium necrophorum* sternoclavicular septic arthritis who uniquely elected for nonsurgical intervention based on her favorable clinical presentation and preference. The patient’s SCSA was managed effectively with intravenous piperacillin + tazobactam with subsequent oral amoxicillin + clavulanic acid. Her nonstandard approach was closely monitored through her long-term recovery. The successful management of this case is presented to expand and broaden the prospective treatment course for SCSA.

## Case presentation

A 38-year-old Caucasian woman with body mass index (BMI, kg/m^2^) of 28.7 kg/m^2^ and medical history notable for anxiety, uterine fibroid treated with fibroid embolization, and human papilloma virus infection was transferred from an outside hospital with neck computed tomography (CT) concerning for cervical abscess and septic arthritis of the left sternoclavicular joint. Four days prior to this presentation, she presented to a local urgent care clinic, where she was tested positive for influenza A and was treated with oseltamivir. While on the therapy, the patient developed acute-onset edema and anterior neck pain, worse upon movement of the neck and the chest. The patient denied any recent trauma, injury, or travel. The patient’s physical examination was notable for diffuse erythema over neck, upper back, and anterior chest wall with tenderness at the left SC joint, bilateral deltoid muscles, and central sternal area. She was afebrile with temperature of 98.7 °F, and laboratory results were unremarkable with white cell count of 18 x 10^9^/L, hemoglobin level of 7.8 g/dl, and blood urea nitrogen level of 4 mg/dL.

The patient underwent emergent IV antibiotic treatment (piperacillin + tazobactam, 3.375 g, Q6H, IV) with surgical exploration, debridement, and drainage, and the intraoperative cultures grew *Fusobacterium necrophorum*. She recovered and was discharged on postoperative day 7 on oral amoxicillin + clavulanic acid (875 mg + 125 mg/day) with close follow-up. At time of discharge, she complained of pain in her left shoulder but magnetic resonance imaging (MRI) demonstrated no evidence of septic joints.

Two weeks after discharge, the patient reported ongoing left shoulder pain upon movement with persistent local edema and tenderness in her left chest. CT scan revealed a new erosion at the left clavicular head and the articular surface of the manubrium at the left sternoclavicular joint, suggestive of new septic arthritis. Her examination was notable for well-healed neck incision and mild erythema and swelling of the left sternoclavicular joint with tenderness. The patient was afebrile, and her laboratory results were notable for erythrocyte sedimentation rate of 85, C-reactive protein level of 10.5, and normal white blood cell count. Metronidazole (500 mg/day) was added to her amoxicillin + clavulanic acid (875 mg + 125 mg/day) antibiotic regimen.

**Fig. 1:**
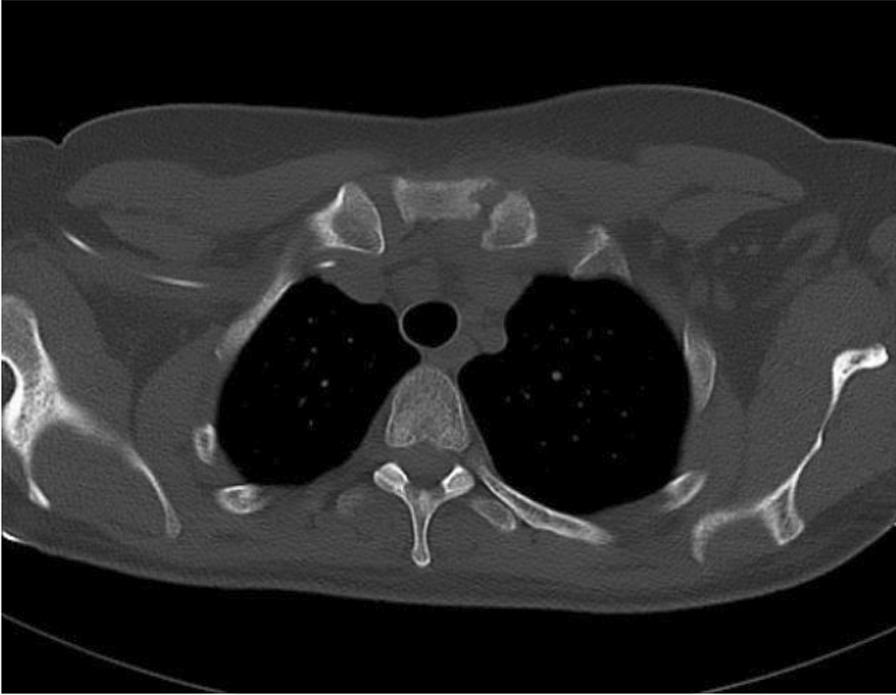
Repeat computed tomography showed increased erosions of the left SC joint

Three weeks after discharge, the patient presented with worsening chest pain, fever, and chills, worsening pain and swelling over her central chest. Repeat CT showed increased erosions of the left sternoclavicular joint (Fig. 1). She was admitted to the hospital, placed on IV antibiotics (pipercillin + tazobactam, 3.375 g, Q6H, IV) and underwent interventional radiology-guided drainage of the left SCSA. She responded well and was discharged with intravenous piperacillin + tazobactam regimen for 7 weeks.

Upon completion of her antibiotic regimen, she was symptom free, with CT showing only minimal residual fat stranding overlying the left pectoralis muscle. The patient was transitioned to sole oral amoxicillin + clavulanic acid treatment for additional 6 weeks. The patient remained well, without symptoms for more than a year.

## Discussion

This case report describes successful nonsurgical management of *Fusobacterium necrophorum*, a rare but potentially relevant pathogen of interest for SCSA. SCSA management is guided by grading of the severity of signs and symptoms. Three weeks after the initial incision and drainage of the SCSA, our patient presented with grade II SCSA (pain and systemic signs of inflammation, joint effusion, and erosion at the sternoclavicular joint), for which surgical treatment is recommended according to Abu *et al*. [12]. However, after careful discussion with the patient and consideration of the overall presentation and health, we opted for successful Interventional-radiology (IR) -guided aspiration and 7 weeks of intravenous piperacillin with peripherally inserted central catheter (PICC) placement (3.375 mg IV q6hr).

This report describes the second case of successful medical management of *F. necrophorum* SCSA. We have identified three variables in our patient that may account for the sufficiency of medical management: the patient’s overall health, the causative organism, and the extent of infection: our patient was young and healthy without risk factors, without osteomyelitis, with *F. necrophorum*. Unlike SCSA caused by other common pathogens such as *Staphylococcus aureus* and *Pseudomonas*, *F. necrophorum* has been demonstrated to respond well to antibiotics [8].

Conservative management with close follow-up should be considered in healthy patients presenting with isolated SCSA caused by certain select pathogens [12]. We suggest careful consideration of treatment options before proceeding with invasive surgical interventions.

## Conclusion

Septic arthritis of sternoclavicular joint is a relatively uncommon infection with significant variations in its categorization and management strategies. This report outlines successful nonstandard nonsurgical management of SCSA caused by rare underlying *F. necrophorum* infection in an otherwise healthy female. We identified three key variables that may be considered regarding medical management of SCSA: patient factors, causative organism, and the extent of infection. We recommend further research and exploration into conservative and multidisciplinary management of isolated *Fusobacterium necrophorum* sternoclavicular septic arthritis in otherwise healthy patients.

## Data Availability

Not applicable.

## Abbreviations

SCSA: Sternoclavicular septic arthritis
MRI: Magnetic resonance imaging
CT: Computed tomography
